# The Impact of Calcination on the Structure, Bioactivity, and Biocompatibility of Sol–Gel-Derived Glasses

**DOI:** 10.1021/acsomega.5c08486

**Published:** 2025-10-28

**Authors:** Renata Nardy Ribeiro, Rosangela Maria Ferreira da Costa e Silva, Rubens Lucas de Freitas Filho, Luiza de Almeida Queiroz Ferreira, Patrick de Souza de Carvalho, Luiz Fernando Cappa de Oliveira, Ivana Márcia Alves Diniz, Walison Arthuso Vasconcellos, Rosana Zacarias Domingues, Ângela Leão Andrade

**Affiliations:** † Departamento de Química, 28115 Universidade Federal de Ouro Preto, Ouro Preto 35400-000, Brasil; ‡ Programa de Pós-Graduação em Recursos Naturais, Universidade Estadual de Mato Grosso do Sul, Dourados 79804-970, Brasil; § Departamento de Química, 28114 Universidade Federal de Minas Gerais, Belo Horizonte 31270-901, Brazil; ∥ Departamento de Odontologia Restauradora, 28114 Universidade Federal de Minas Gerais, Belo Horizonte 31270-901, Brasil; ⊥ Departamento de Química, 28113 Universidade Federal de Juiz de Fora, Juiz de Fora 36036-900, Brazil

## Abstract

Bioactive glasses (BG) are promising materials for bone tissue engineering due to their bonding ability to biological tissues. In this study, a BG was synthesized via a modified sol–gel method that incorporated fumed silica. Two thermal treatments were applied: stabilization at 36.5 °C and calcination at 700 °C. X-ray diffraction analysis revealed the amorphous nature of BG stabilized at 36.5 °C, while calcination induced the formation of crystalline calcite. Nitrogen adsorption–desorption analysis revealed a specific surface area of 62 m^2^ g^–1^ for the stabilized BG, which decreased to 32 m^2^ g^–1^ after calcination. Thermogravimetric analysis indicated that the calcined sample exhibited lower thermal stability than the stabilized sample. Both BG samples exhibited the capacity to form a hydroxyapatite layer upon immersion in a simulated body fluid for a period of 48 h, being corroborated by X-ray diffraction, FTIR, and scanning electron microscopy. Additionally, cell viability assays with HaCaT keratinocytes showed enhanced cell proliferation compared to the control at 1, 4, and 7 days. A toothpaste containing 3 w/w% BG was also formulated with the calcinated sample and tested for dentin hypersensitivity, showing promising dentinal tubule occlusion, remineralization potential, and cytocompatibility. These results indicate that both samples prepared retains excellent surface properties, bioactivity, and cytocompatibility, even without high-temperature processing, highlighting its potential for biomedical applications.

## Graphical abstract

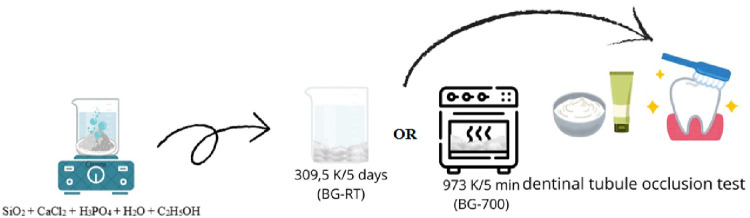

## 1 Introduction

Due to the limited availability of autografts and allografts, the repairing of bone defects caused by trauma, disease and other pathological conditions remains a significant clinical challenge.^1,2^ This scarcity has driven the development of alternative biomaterials capable of supporting bone regeneration. Among the most studied materials, bioactive glasses (BGs) have emerged as promising candidates.^2^ BGs are primarily composed of silicon, phosphorus, and calcium oxides and were first introduced by Hench and Polak in 1971.^3^

BGs are distinguished by their ability to form a hydroxycarbonate apatite (HCA) layer when in contact with physiological fluids such as simulated body fluid (SBF).^4–6^ This apatite layer mimics the mineral phase of bone and enables strong bonding between the material and host tissue.^7,8^ Due to their excellent bioactivity, biocompatibility, osteoconductivity, and bioresorbability, BGs have been widely applied in orthopedic, dental, and maxillofacial surgeries for the restoration and regeneration of damaged or diseased bone.^9,10^

There are two main synthesis routes that are commonly utilized for the production of BG. The traditional melt-quenching method was first employed. Later, the sol–gel process was developed as a mild alternative process. While the melt-quenching method involves the high-temperature processing of oxide precursors, this may result in the loss of volatile components such as P_2_O_5_ and limit the incorporation of heat-sensitive biomolecules.^11,12^ In this regard, the sol–gel method offers several advantages, including lower synthesis temperatures, thereby enabling enhanced control over their composition and structure.^13^ Sol–gel-derived glasses typically exhibit higher specific surface areas^13,14^ and a greater number of silanol (Si-OH) surface groups, which significantly enhance their bioactivity and apatite-forming ability. This method also allows for the production of BGs with tunable degradation rates and mechanical properties.^15^

Despite the advantages inherent in the sol–gel method, there are challenges associated with its implementation. These include the necessity of calcination in order to remove residual organics^8^ and the use of some potential toxic chemical precursors.^16^ In response to these limitations, alternative approaches and precursors have been explored to improve the biocompatibility and sustainability of the synthesis process.

Recent studies have raised concerns regarding the cytotoxicity of certain forms of silica-based materials, such as pyrogenic silica and nanoquartz, due to their highly reactive surfaces. Nevertheless, the employment of modified silica sources and more environmentally friendly synthesis pathways has the potential to alleviate these concerns and augment the safety profile of bioactive glasses.^17,18^

Dentine hypersensitivity is characterized by acute, short-lasting pain arising from exposed dentine in response to thermal, evaporative, tactile, osmotic or chemical stimuli, in the absence of other dental defects or pathologies, being associated with the presence of open dentinal tubules on the dentinal surface.^19,20^ The most widely accepted explanation for its mechanism is the “hydrodynamic theory,” proposed by Brännström in 1963, which attributes the pain to fluid movement within open dentinal tubules that indirectly stimulates pulp nerves.^21,22^ In support of this theory, hypersensitive dentine has been observed to exhibit tubules that are wider and more numerous than those on nonsensitive surfaces. The latter are typically occluded by a smear layer.^22–24^

Strategies for reducing sensitivity often involve mechanical or chemical occlusion of these tubules, with active treatments showing superior outcomes compared to placebo.^25–27^ Among them, nanohydroxyapatite (n-HAp) toothpaste has shown promising effects in alleviating both dentin hypersensitivity and postbleaching sensitivity.^28,29^ Apatite crystals exhibit both morphological and structural similarity to natural enamel, thus rendering n-HAp nanoparticles suitable as biomimetic agents, i.e., capable of promoting remineralization in enamel affected by mineral loss.^30,31^

This study reports the synthesis of a novel bioactive glass via a modified sol–gel method using pyrogenic silica, enabling precise control of composition and surface properties. Part of the glass was stabilized at 36.5 °C and part calcined at 700 °C. While both exhibited bioactivity in SBF, only the calcined glass was incorporated into a toothpaste formulation and evaluated for its biological performance. Specifically, this study investigates the *in vitro* bioactivity of BG and its potential to occlude dentinal tubules in human teeth, addressing dentin hypersensitivity. By bridging material development with practical application in a functional dental care product, this work provides a distinctive and translational contribution, highlighting a novel route for the management of dentin hypersensitivity.

## 2 Experimental Section

### 2.1 Materials

All chemicals used in this work were of analytical grade and used as received. The composition of the mixture includes commercial pyrogenic silica (SiO_2_) (Evonik), phosphoric acid (H_3_PO_4_, 85%) (Panreac), calcium chloride dihydrate (CaCl_2_·2H_2_O, 99%) (Sigma-Aldrich), absolute ethanol (C_2_H_5_OH, 99.5%) (Panreac), Sensodyne Rápido alívio, sorbitol (C_6_H_1__4_O_6_) (Synth), glycerol (C_3_H_8_O_3_) (Synth), potassium sorbate (C_6_H_7_KO_2_) (Synth), carboxymethyl cellulose {[C_6_H_7_O_2_(OH)_*x*_(OCH_2_COONa)_*y*_]_*n*_} (Synth), titanium dioxide (TiO_2_) (Synth), sodium lauryl sulfate (C_1__2_H_2__5_NaO_4_S) (Cloroquímica; Maian).

### 2.2 Synthesis Bioactive Glass

BG with theoretical composition 61SiO_2_–37CaO–2P_2_O_5_ (mol %) was synthesized through a modified sol–gel process. In the beginning, 0.016 mol of H_3_PO_4_ (as the phosphorus source) was added to 65 mL of deionized water and subsequently stirred for 2 min. 212 mL ethanol was then introduced into the solution to facilitate dispersion of the components and promote rapid volatilization during the sol–gel formation process. The mixture was stirred for an additional period of 2 min. The commercial pyrogenic silica was then incorporated (as the silica source). The molar ratio of the sol was 16:1 (ethanol:SiO_2_). The solution was magnetically stirred to 1 h at room temperature.

Subsequently, 0.13 mol CaCl_2_·2H_2_O was gently added to the solution (as the calcium source). The sol was subjected to continuous stirring for an additional period, ceasing once the gelation process impeded the stirring process. After gelation, the gel was kept under a humidified atmosphere inside an amber bottle for 5 days, and was moistened with acetone. The sample underwent a series of gentle washes with deionized water, performed in a controlled manner to efficiently remove chloride while preserving the overall composition of the material. The washed gel was divided into two portions that underwent different heat treatments: (i) drying in an oven at 36.5 °C for 5 days. This sample were labeled as BG-RT; (ii) calcined at 700 °C for a duration of 5 min. This sample were labeled as BG-700.

### 2.3 Assessment of *In Vitro* Bioactivity

Bioactivity tests were performed by soaking the particles BG-RT and BG-700 in SBF at 37 °C (body temperature), at a concentration of 1 mg mL^–1^, during 24, 48, and 72 h. The SBF was prepared as described previously^32^ yielding ionic concentrations of (in mmol L^–1^): 142.0 Na^+^, 5.0 K^+^, 2.5 Ca^2+^, 1.5 Mg^2+^, 4.2 HCO_3_^–^, 148 Cl^–^, 1.0 HPO_4_^2–^, and 0.5 SO_4_^2–^ and pH = 7.4 at 37 °C. Throughout the duration of the experiment, the existing solution was entirely extracted and substituted with a freshly prepared solution at 48 h intervals. Subsequently, the particles were rinsed with deionized water subsequently analyzed for the nucleation of calcium phosphate.

### 2.4 Bioactive Glass Dental Paste: Preparation and Use

#### 2.4.1 Preparation of the Experimental Toothpaste

The experimental toothpaste was prepared in three distinct steps (S1–S3), following a carefully controlled sequential addition of ingredients. In each step, the ingredients were mixed under hygienic conditions using deionized water heated to 55 °C (when indicated). The complete toothpaste formulation is summarized in Table 1.

**Table 1:** Stepwise and Overall Composition of Toothpaste^a^

	percentage (% w/w)		
ingredient	S1	S2	S3
sorbitol	6.42 (1)	2.58 (1)	8.83 (1)
glycerin	3.17 (2)	5.08 (2)	16.67 (3)
carboxymethylcellulose (CMC)	1.08 (3)		
deionized water (55 °C)	16.67 (4)		22.92 (4)
potassium sorbate	0.18 (5)		
titanium dioxide		1.31 (3)	
fumed silica			5.00 (2)
sodium lauryl sulfate			1.00 (5)
sucralose solution (1%)			0.33 (6)
menthyl lactate			0.50 (7)

a Numbers in parentheses indicate the order of ingredient addition in each phase.

The S1 components were added sequentially according to the order indicated. After each addition, the mixture was homogenized until complete dissolution before the next component was introduced. The final mixture was left undisturbed for 12 h to allow complete hydration and stabilization of the gel matrix.

For S2, the ingredients were incorporated in the specified order but vigorously mixed until each component was fully solubilized. The homogeneous mixture was also allowed to rest for 12 h to ensure full hydration.

Once fully hydrated, S1 and S2 were mixed by pouring S1 into S2 under continuous stirring to form a uniform base. The ingredients of S3 were then incorporated into the combined base (S1 + S2) in the given order. Each component was thoroughly mixed until complete dispersion or dissolution before the next ingredient was added. The final mixture was homogenized until smooth and deionized water was added to adjust the total batch weight to 1200 g of toothpaste. At the end of the preparation process, 3% (w/w) of the of the calcined synthesized BG (BG-700) was incorporated into the formulation and thoroughly blended to ensure homogeneous distribution throughout the final product.

#### 2.4.2 Demineralization of Bovine Tooth

Previously extracted caries-free bovine incisors were obtained from a certified tooth bank. Dentin square samples (4 mm × 4 mm) with approximately 1.0 mm thickness were prepared by cutting perpendicularly to the long axis of the tooth 4 mm above the cemento-enamel junction, using a diamond blade (Buehler, Lake Bluff, IL, USA). Dentin lesions were induced by acid-etching with 37% phosphoric acid for 20 s to promote controlled demineralization.^33^ The resulting demineralized specimens were gently rinsed under running distilled water for 30 s to remove any residual debris and subsequently immersed in artificial saliva to initiate the experimental procedure.

#### 2.4.3 Immersion Protocol Following Toothpaste Application

The demineralized dentin specimens were randomly assigned to three experimental groups (*n* = 3 per group), as described below. Samples were collected after 2 and 4 consecutive days of treatment. The composition of artificial saliva (pH 7.4) consists of CaCl_2_ 1.5 mmol L^–1^, KCl 50 mmol L^–1^, KH_2_PO_4_ 0.9 mmol L^–1^, and Tris 20 mmol L^–1^.^34^

Group I—Negative Control (Artificial Saliva Only): each demineralized dentin square was immersed in 20 mL of artificial saliva, which was refreshed three times daily—mornings, afternoons and evenings and were stored at 37 °C. No active treatment was applied to the specimens of this negative control group.

Group II—Experimental Toothpaste (BG): the experimental toothpaste formulated in this study containing BG was applied (20 mg) using a sterile cotton swab. Each demineralized dentin square received a gentle application of the paste for 1 min, three times a day (mornings, afternoons, and evenings). After each application, specimens were rinsed under running distilled water for 1 min to remove any residual product, then immersed in 20 mL of fresh artificial saliva (pH 7.4), and stored at 37 °C. The artificial saliva was replaced after every brushing session.

Group III—Commercial Desensitizing Toothpaste (Sensodyne Rápido Alívio): this group followed the same treatment protocol as Group II, using a commercially available desensitizing toothpaste (Sensodyne Rápido Alívio) instead of the experimental formulation. Brushing, rinsing, and artificial saliva immersion procedures were carried out with the same frequency for up 4 days.

In all groups, demineralized dentin squares were kept in artificial saliva for periods of 2 and 4 days. At the end of the immersion period, samples were prepared for further morphological and/or physicochemical analysis.

### 2.5 Biological Evaluation

#### 2.5.1 Cell Culture

Human keratinocytes (HaCat) were expanded in supplemented culture medium Dulbecco’s Modified Essential Medium - DMEM, with 10% fetal bovine serum (FBS) and 1% penicillin/streptomycin (all from GIBCO). Then, cells were plated at 1 × 10^4^ cells/well in quadruplicate in 48-wells microplates and after 24 h, the conditioned medium was added to the cultures. Upon in contact with the cell cultures, the medium was refreshed every 2 days.

#### 2.5.2 Biological Assays

BG-RT and BG-700 ion extracts were prepared as previously described.^35^ Briefly, 1% w/v of each powder was soaked in supplemented *N*,*N*-dimethylethylenediamine (DMEN). Prior to the addition of further components to the cultures, the mixture was subjected to a 24 h incubation period within a humidified incubator maintained at a temperature of 37 °C and a CO_2_ concentration of 5% (Binder, Tuttlingen, Germany). The supernatant was then collected and filtered (0.22 μm).

In order to measure the cell viability of HaCat cells in contact with BG-RT and BG-700 ions extracts, the 3-(4,5-dimethylthiazol-2-yl)-2,5-diphenyl-tetrazolium bromide (MTT) reduction assay (Vybrant MTT Cell Proliferation Assay Kit, Invitrogen) was performed in accordance with the manufacturer’s guidelines. Briefly, after 1, 4, and 7 days of cell culturing, the MTT solution was added to the cultures, and cells were incubated for 2 h at 37 °C in the presence of 5% CO_2_. The medium was then removed and formazan crystals formed during this process were extracted by adding dimethyl sulfoxide (DMSO) (Sigma-Aldrich). After 15 min, the absorbance was read at 540 nm in a microplate reader (BioTek Instruments, Biochrom Ltd., Eugendorf, Austria).

### 2.6 Characterization Techniques

The phase constituents of BGs and hydroxyapatite were evaluated with XRD (Bruker Company, USA) using the Cu Kα radiation (1.54184 A°) operating at 30 kV and 10 mA, scanning range from 7 to 70 °C and a step size of 0.02 °, 1 s per step. FTIR spectra of the powders were obtained using Perkin-Elmer Spectrum GX spectrophotometer. The BG powders and potassium bromide were blended at a ratio of approximately 1%. The spectra of transmittance mode were examined in the 400–4000 cm^–1^ range by the resolution of 4 cm^–1^.

The nitrogen adsorption/desorption analyses were conducted utilizing an Autosorb iQ equipment (Quantachrome Instruments, USA) at −196 °C in the relative pressure range of 0.005–1.0, with those previously degassed at 150 °C for 12 h under vacuum conditions. The surface area of each material was estimated by the BET method (Brunauer, Emmett, Teller), the pore size distribution was estimated by the BJH method (Barrett–Joyner–Halenda) and the total pore volume was measured at the relative pressure of 0.99. The acquisition of experimental data and data processing were obtained using the ASiQwin 5.21 software version. The TG and DTG curves were obtained in a Shimadzu Simultaneous TGA/DTA Analyzer DTG-60H equipment. The analyzes were carried out in an alumina crucible and the following furnace settings were used: heating ratio of 10 °C·min^–1^, starting from room temperature up to 900 °C, in an air atmosphere with a flow of 50 mL·min^–1^. Weights of BG samples were around 2–5 mg.

To evaluate the progression of dental treatment following artificial saliva immersion, scanning electron microscopy (SEM) was performed using a FEI Quanta 250 microscope operating in low-vacuum mode (1 mbar) at 5000× magnification. Representative areas from each sample were systematically imaged to assess morphological changes. Raman spectra were recorded in a Bruker SENTERRA spectrometer with 632.8 nm exciting radiation, with laser power of 10 mW at the sample, 10 cycles of 25 s each and spectral resolution of 3–5 cm^–1^. The Raman spectra were obtained at least twice to guarantee the wavenumber and intensity of each one of the bands observed at the spectra, being all spectra subjected to analysis using Origin 8.0.

### 2.7 Statistical Analysis

The Shapiro-Wilk test was performed to evaluate data distribution. All data were analyzed using the ANOVA, followed by Tukey post hoc, at a significance level of 5%. This analysis was performed using GraphPad Prism 9 (GraphPad Software Inc., La Jolla, CA, USA; https://www.graphpad.com).

## 3 Results and Discussion

### 3.1 Characterization of the Synthesized Samples

Figure 1 shows the FTIR of the pure silica, BG-RT and BG-700 samples. Pure silica exhibits a band at ∼450 cm^–1^, which can be assigned to γ­(Si–O–Si), corresponding to out-of-plane bending of the silicate network. For BG-RT and BG-700 samples, this band is shift to 470 cm^–1^. Some authors have shown that the presence of cations may also be the cause of the shift to higher frequency of the Si–O–Si band maximum.^36,37^ The absorption bands in the 1100–1200 cm^–1^ range, as well as around 800 cm^–1^, are attributable to ν­(Si–O–Si) stretching vibrations within the gel network.^38–40^ A band at ∼960 cm^–1^ observed in pure silica is assigned to (ν­(Si–OH)) and may be related to the formation of silanol (Si–OH) groups.^41^ It is observed that when the glass samples are dried at 36.5 and 700 °C, this band decreases in intensity since thermal treatment induces sample dehydration (through condensation of surface hydroxyls: 2SiOH → SiOSi + H_2_O), that should be related to the lower amount of hydroxyl groups.

**Figure 1:**
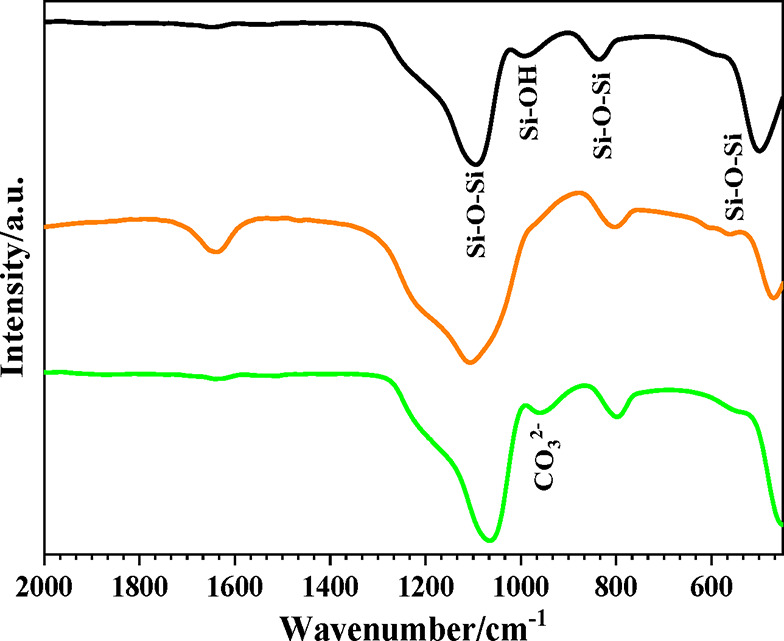
FTIR spectra of samples: (a) pyrogenic silica, (b) BG-RT, and (c) BG-700.

The band within the region of 1200–900 cm^–1^ is attributable to both PO and SiO groups. As for the SiO groups, these can be grouped as SiO ‘bridging’ and ‘nonbridging’ to other SiO_4_ tetrahedra. Indeed, a proportion of the oxygen atoms are not directly connected to another silicon atom within the glassy network. This is due to the presence of modifier cations, which induce the formation of ‘nonbridging’ SiO groups.^42^ Therefore, the presence of PO bonds and Ca^2+^ in the BG-RT and BG-700 samples may justify the widening and displacement of the band to 1000 cm^–1^, if compared to the spectrum of pure silica.

In contrast to BG-RT, BG-700 sample exhibits a distinct band near 978 cm^–1^, corresponding to ν­(CO_3_^2–^) in calcite, as shown in Figure 1c.^43^ This phenomenon is well supported by the literature. Li et al. demonstrated that calcium-containing silicate glasses are highly reactive toward atmospheric CO_2_, especially those with higher Ca/Si ratios, leading to the formation of CaCO_3_ detectable by FTIR.^44^ Similarly, Kalinkin et al. reported that CaO readily reacts with CO_2_ at elevated calcination temperatures to form stable calcium carbonate phases.^45^ These findings strongly support our interpretation that the carbonate band observed in the BG-700 sample originated from a secondary reaction between CaO and CO_2_ during or after calcination, rather than from carbonate incorporation during the initial synthesis.

Powder X-ray diffraction (XRD) patterns for the synthesized samples, BG-RT and BG-700, are shown in the Figure 2. A broad band in the range of 20°–34° (2θ) could be ascribed to the amorphous silicate while no diffraction peaks could be observed in the XRD patterns indicating the amorphous nature of BG-RT sample. After heating to 700 °C (BG-700 sample), it can be observed a crystalline structure with sharp reflection peaks at 2θ = 23.04°, 29.44°, 31.48°, 36.00°, 39.44°, 43.15°, 47.12, 47.49°, 48.51°, 56.55°, 57.40°, 60.68°, 60.98, 63.06°, 64.68°, 65.60°, 69.23°, corresponding to the diffraction of the crystal planes of calcite-syn 012, 104, 006, 110, 113, 202, 024, 018, 116, 211, 122, 214, 208, 125, 300, 0012, 217, respectively (ICDD # 5-586). This phase confirms the emergence of the CO_3_^2–^ peak in the BG-700 sample, as observed in the FTIR spectrum.

**Figure 2:**
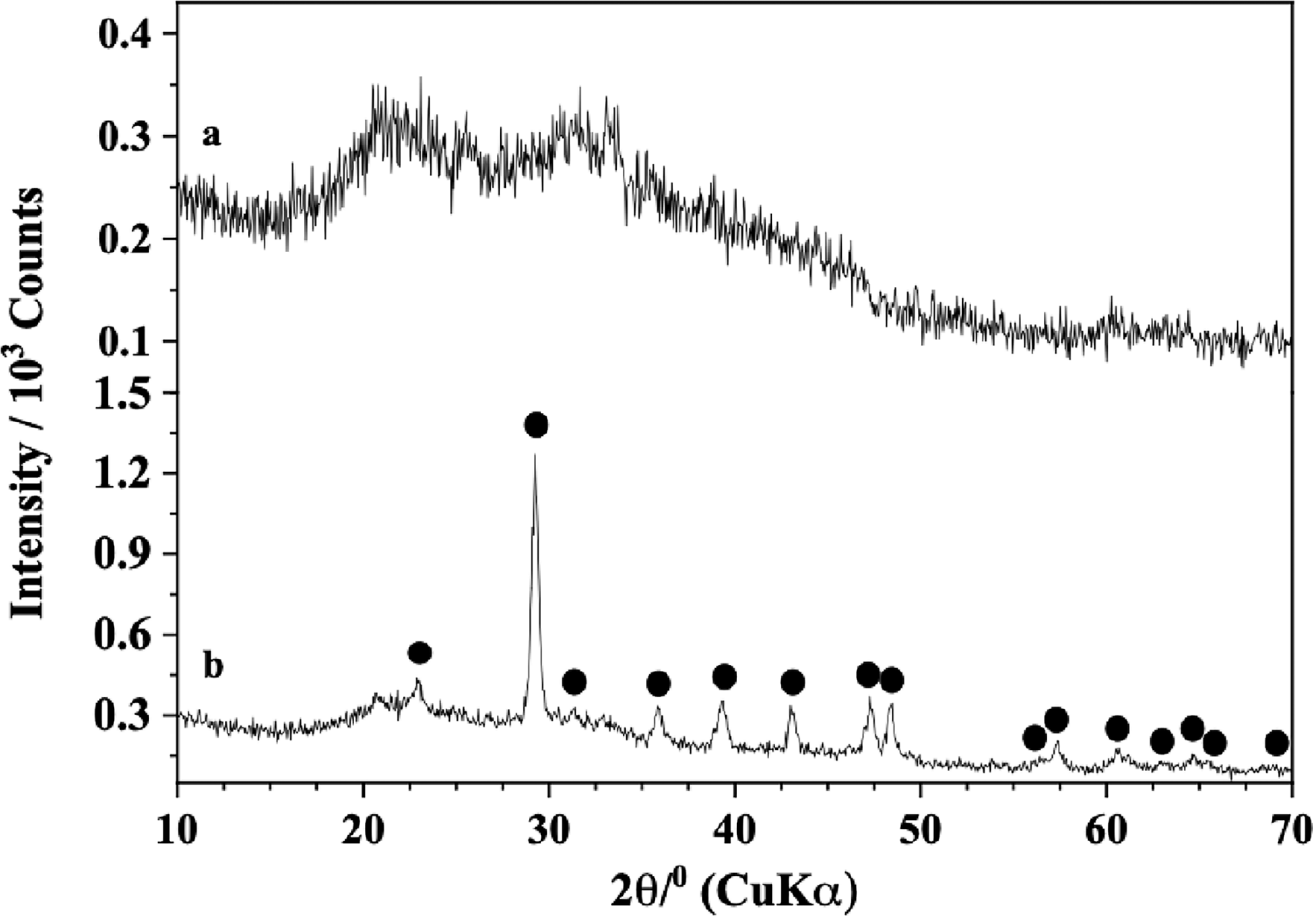
Powder X-ray diffraction patterns of the samples: (a) BG-RT and (b) BG-700.

Nitrogen adsorption–desorption analyses at 77 K were performed to compare the specific surface area (*S*_BET_) and porosity of BG-RT and BG-700. As illustrated in Figure 3a, both samples exhibited type IV isotherms, a characteristic of mesoporous materials, and H1-type hysteresis loops, indicative of an open-pore network.^46,47^ The N_2_ adsorption–desorption isotherm and pore size distributions (Figure 3b) show that calcination at 700 °C led to a pronounced reduction in textural parameters, with the specific surface area decreasing by approximately 50%, accompanied by reductions in total pore volume and average mesopore diameter (Table 2). Similar trends were reported by Wajda and Sitarz,^48^ who synthesized CaO–SiO_2_ bioactive glasses via sol–gel and melting techniques. Their sol–gel-derived BGs presented *S*_BET_ = 136 m^2^ g^–1^, mesopore volume (BJH) = 0.401 cm^3^ g^–1^, and average pore diameter (BJH) = 8.58 nm.

**Figure 3:**
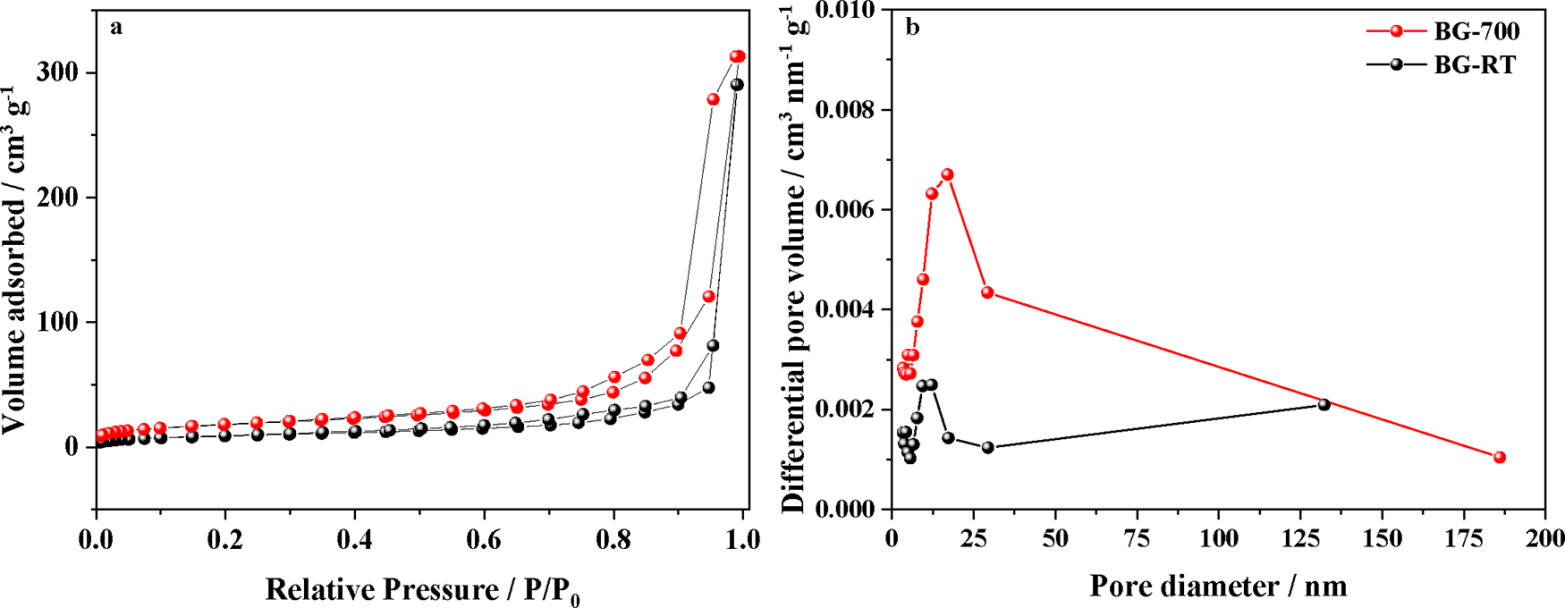
: (a) N_2_ adsorption–desorption isotherms at 77 K and (b) pore size distribution of mesoporous in the samples BG-RT and BG-700.

**Table 2:** Textural Parameters by BET and BJH Method from BG-RT and BG-700

sample	*S*_BET_/m^2^ g^–1^	*S*_meso_ ^a^/m^2^ g^–1^	*V*_meso_ ^a^/cm^3^ g^–1^	*V*_tp_ ^b^/cm^3^ g^–1^	*D*_p_ ^a^/nm
BG-RT	64	50	0.476	0.484	17.02
BG-700	32	27	0.446	0.449	12.12

a Calculated using the BJH method.

b Total pore volume at *p*/*p*_0_ = 0.994; *D*_p_ = average pore diameter by BJH method.

Schumacher et al.^47^ synthesized a series of mesoporous bioactive glasses (MBGs) using different SiO_2_:CaO:P_2_O_5_ ratios and calcined at 600 °C. The specific surface areas ranged from 309 to 630 m^2^ g^–1^, with pore sizes in the range of 3.76–5.91 nm. Similarly, Yan et al.^49^ produced MBGs with varying compositions and investigated the effect of calcination temperatures between 500 and 900 °C. In the case of the series calcined at 700 °C, the specific surface area was found to range from 300 to 350 m^2^ g^–1^, the total pore volume from 0.43 to 0.49 cm^3^ g^–1^, and the average pore diameter from 5.0 to 5.6 nm. Although the surface areas obtained in the present work are lower, the mesopore volumes are comparable and the pore size distribution and average diameters measured here are substantially higher than those reported in the present literature.^47–49^

The TG thermograms of the BG-RT and BG-700 samples are shown in Figure 4.

**Figure 4:**
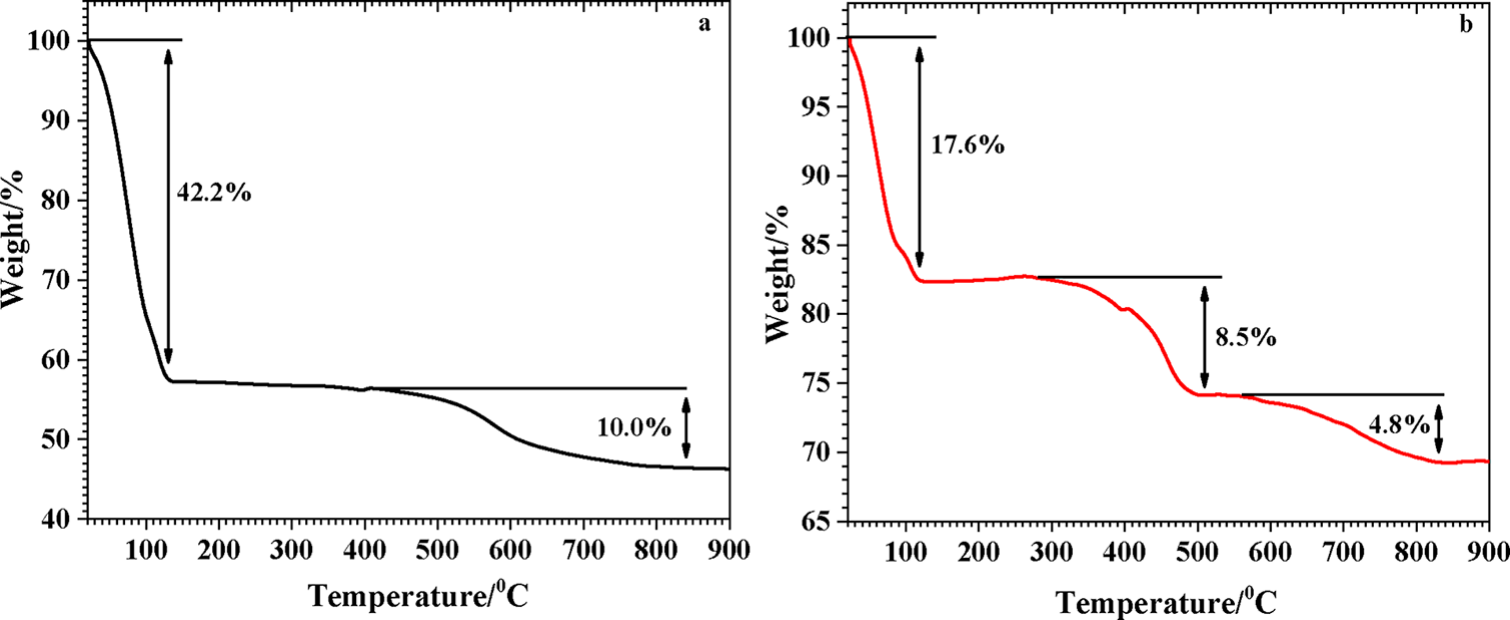
TGA curves for (a) BG-RT and (b) BG-700.

The thermogravimetric analysis (TGA) curve of BG-RT (Figure 4a) exhibited a large mass loss of 42.2% between ∼20 and 130 °C, attributable to physiosorbed water and residual solvents.^50^ A second broad loss of 10.0% occurred between 420 and 840 °C, consistent with a progressive dehydroxylation of surface Si–OH groups, forming siloxane (−Si–O–Si−) bridges and releasing water.^51^ After thermal treatment at 700 °C (BG-700 sample) (Figure 4b), the low-temperature loss was reduced to 17.6% (20–130 °C), suggesting the elimination of the majority of physosorbed species; two additional mass-loss regions (≈280–510 °C, 8.5% and 560–830 °C, 4.8%) are assigned to final dehydroxylation/condensation of the silica network and the decomposition of carbonate species with release of CO_2_, respectively.^52,53^

The distinct thermogravimetric behaviors of BG-RT and BG-700 can be rationalized by differences in their chemistry surface and textural properties. The synthesized from fumed silica and maintained at 36.5 °C BG-RT sample exhibited a markedly high initial mass loss (42.2% between ∼20 and 130 °C), reflecting its large BET surface area and, consequently, its greater capacity to accommodate physisorbed water. This behavior is consistent with the abundance of accessible silanol groups and the open surface topology of the low-temperature material, which favors the adsorption of water molecules. In contrast, the BG-700 sample obtained after thermal treatment at 700 °C displayed a substantially reduced low-temperature loss (17.6%), attributable to the removal of most physisorbed species and partial collapse or coarsening of the porous network, leading to a lower specific surface area. The additional mass-loss step observed in BG-700 at intermediate (≈280–510 °C, 8.5%) temperatures correspond to the final stages of dehydroxylation and siloxane bridge formation within the densified silica framework. These differences illustrate how thermal history modulates the balance between physisorbed and structurally bound water, as well as the energetics of water release from silica-based glass surfaces.^54–56^

The application of thermal treatment at a temperature of 700 °C resulted in a shift in all of the decomposition peaks of BG-RT sample toward lower temperatures (Figure 5), suggesting a decrease in chemical stability relative to its untreated counterpart. This reduced stability can be attributed to the presence of carbonate species detected in the BG-700 sample, at least in part, as evidenced by the characteristic FTIR band near 876 cm^–1^ and the clear identification of calcite via XRD. The decomposition of these carbonate phases typically occurs at temperatures between 650 and 850 °C, contributing to the observed mass loss and affecting the overall thermal behavior. Similar shifts in decomposition temperatures and the influence of carbonate presence have been reported in studies of BGs and silica-based materials subjected to thermal treatments, therefore corroborating our findings.^53^

**Figure 5:**
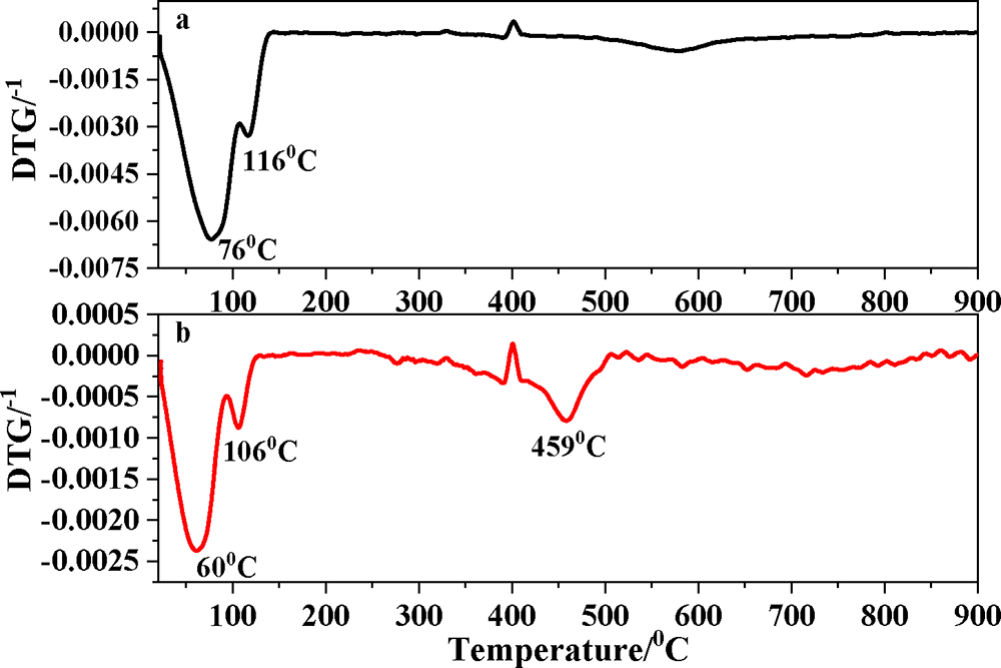
Comparison of DTGA curves for: (a) BG-RT and (b) BG-700 samples.

### 3.2 *In Vitro* Bioactivity Testing in SBF

The kinetics of hydroxyapatite formation onto the surfaces of BG-RT and BG-700 samples were monitored using XRD and FTIR measurements. Figure 6a,b display the X-ray diffraction patterns for BG-RT and BG-700 samples, respectively. Prior to immersion in SBF, BG-RT sample exhibited a broad XRD band between 20° and 40° (2θ), which is characteristic of an amorphous silicate structure^57^ (Figure 6a 0 h SBF). After 24 h of immersion in SBF, two new broad diffraction peaks emerged, corresponding to the (002) and (211) planes of an apatite-like phase (ICDD card #1-1008). With increasing immersion time, the most intense diffraction peak became progressively sharper and better defined, indicating the growth of a more crystalline apatite phase onto the glass surface.

**Figure 6:**
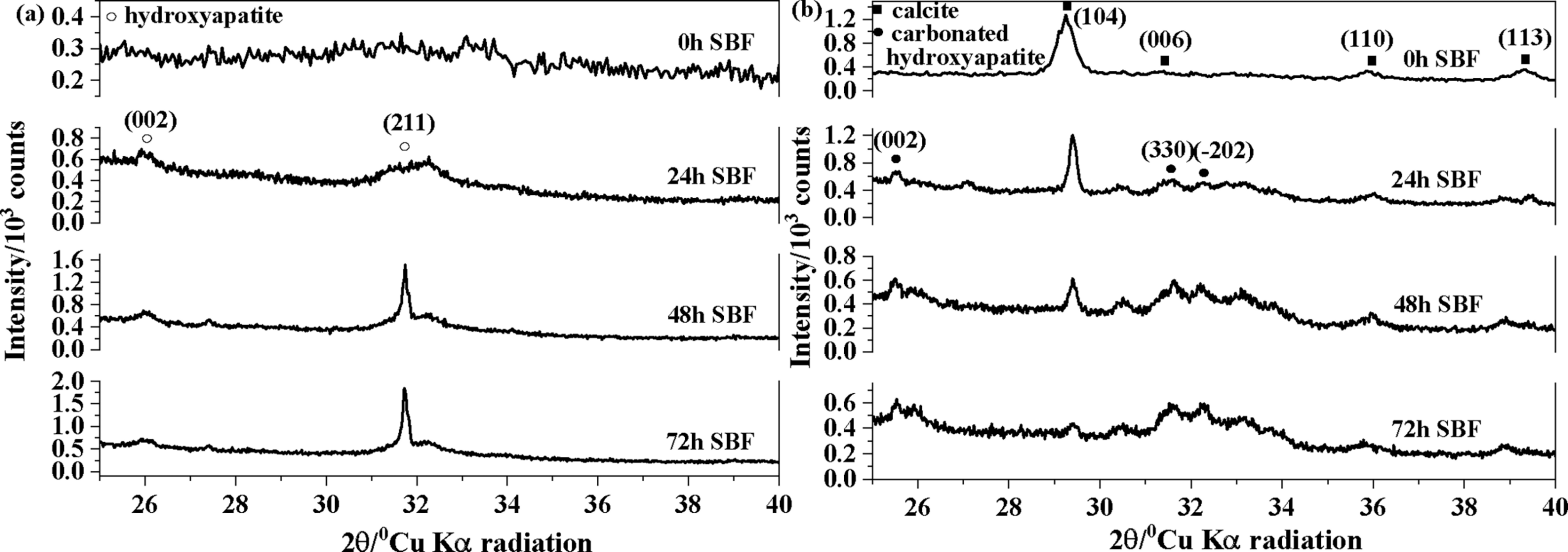
X-ray diffraction patterns of (a) BG-RT and (b) BG-700 samples, before and after immersion in SBF for 24, 48, and 72 h.

The observation that only the (211) reflection appears as a well-defined and sharp peak, while other reflections, such as (002), remain broad can be attributed to differences in the crystallization dynamics of the hydroxyapatite phase during nucleation and growth in SBF. The (211) plane is the most intense and characteristic reflection of hydroxyapatite (ICDD card #1-1008), corresponding to a direction of higher structural stability and lower surface energy, which promotes the preferential growth of larger and more ordered crystallites along this orientation. As a result, the (211) peak becomes progressively sharper and more intense with increasing immersion time. In contrast, other planes such as (002), may retain higher defect densities, residual lattice strain, or partial incorporation of amorphous residues from the glass matrix, which hinder complete crystallization and lead to peak broadening. Similar behaviors have been reported for bioactive glasses and biomimetic hydroxyapatites, where the early stages of apatite nucleation often result in anisotropic growth with a dominant (211) reflection, followed by slower structural ordering along other crystallographic planes.^58–60^

In the case of BG-700 sample, even before immersion in SBF, well-defined diffraction peaks were observed, which were attributed to a crystalline calcite phase, corresponding to the (104), (006), (110), and (113) reflections (ICDD card # 5-586) (Figure 6b 0 h SBF). After 24 h of immersion in SBF, additional peaks were detected and assigned to a carbonated apatite-like phase (ICDD card #35-180). This indicates the nucleation and initial growth of a bioactive layer on the glass surface, consistent with the early stages of bioactive glass mineralization reported in the literature.^61,62^

The difference in the crystalline phases formed onto BG-RT and BG-700 samples after immersion in SBF can be attributed to their initial structural and compositional characteristics. The BG-RT sample, which was fully amorphous before immersion, provided a homogeneous silicate matrix that favored the nucleation and growth of stoichiometric hydroxyapatite. In contrast, the BG-700 sample initially contained a crystalline calcite phase, as confirmed by its diffraction pattern prior to immersion. Calcite is known to release carbonate ions (CO_3_^2–^) upon partial dissolution in aqueous environments, especially under the slightly alkaline conditions of SBF. These carbonate ions can become incorporated into the forming apatite lattice by substituting either phosphate (B-type substitution) or hydroxyl (A-type substitution) sites, resulting in the precipitation of carbonated apatite rather than pure hydroxyapatite. This process is thermodynamically favored because the presence of carbonate reduces the activation energy for nucleation, thereby directing the crystallization pathway toward a carbonated apatite structure. Similar behavior has been reported in BG systems containing pre-existing calcium carbonate phases, where carbonate release during immersion plays a decisive role in determining the final apatite composition.^63,64^

Figure 7 presents the FTIR spectra of BG-RT and BG-700 samples, both in their as-prepared state and after immersion in SBF for 24, 48, and 72 h. In the as-prepared BG-RT sample, characteristic bands associated with silicate groups are observed: a band near 470 cm^–1^ (γ­(Si–O–Si)), a broad envelope between 1100 and 1200 cm^–1^ (ν­(Si–O–Si)), and a peak at approximately 800 cm^–1^ (ν­(Si–O–Si), corresponding to Si–O–Si stretching vibrations.^36–40^ Additionally, a distinct band at ∼960 cm^–1^ (ν­(Si–OH)) is observed, indicating the presence of silanol groups on the glass surface.^41^

**Figure 7:**
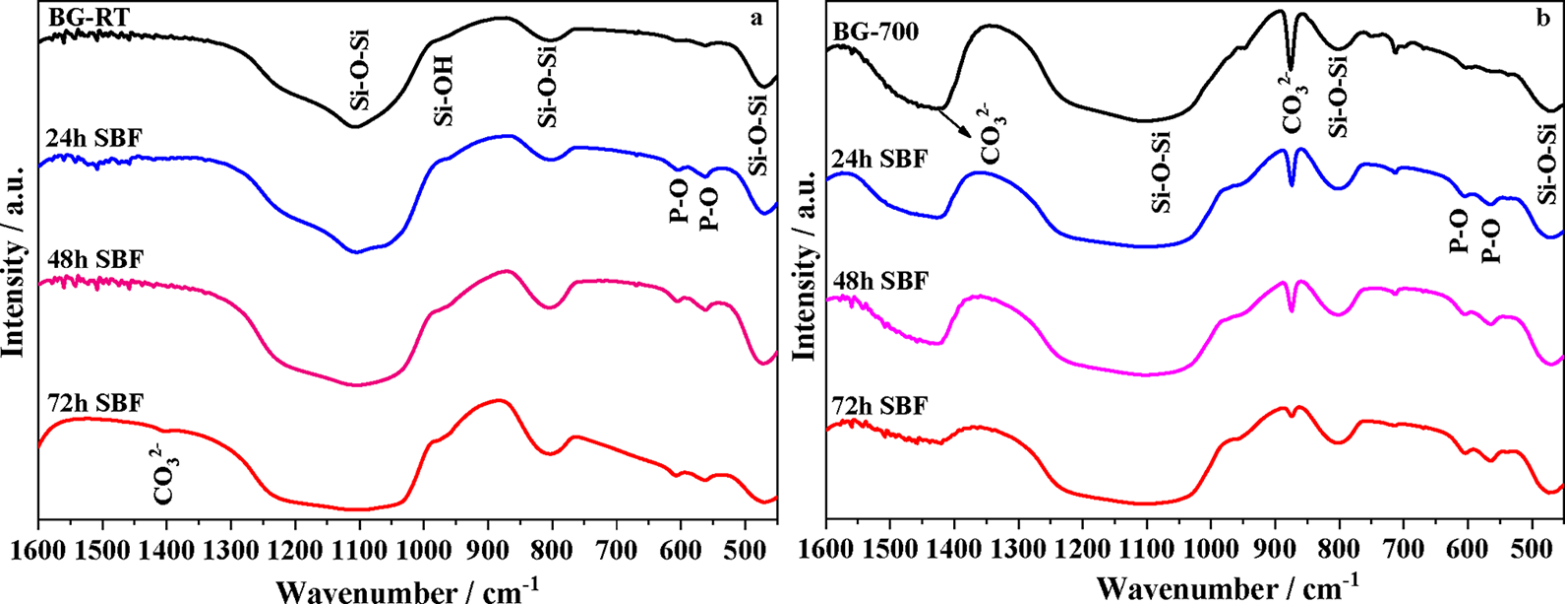
FTIR spectra of (a) BG-RT and (b) BG-700 samples before and after immersion in SBF for 24, 48, and 72 h.

In contrast, the FTIR spectrum of BG-700 sample not only displays the typical Si–O–Si bands but also exhibits additional features indicative of carbonate-containing phases. A prominent band at 876 cm^–1^ (ν­(CO_3_^2–^) is assigned to calcite,^43,65^ while a band at 1423 cm^–1^ corresponds to B-type carbonate substitution in apatite, where carbonate ions replace phosphate groups within the hydroxyapatite lattice.^57^

A comparison of the FTIR spectra of BG-700 before and after immersion in SBF (Figure 6) reveals that, after 24 h of immersion, new transmittance bands emerge at 560 and 600 cm^–1^ (ν_4_(PO_4_^3–^) in both BG-RT and BG-700 spectra, characteristic of phosphate groups and consistent with hydroxyapatite formation.^66^ With increasing immersion time, the intensity of these phosphate bands progressively increases, indicating continuous nucleation and growth of the apatite phase on the glass surface. This evolution is directly linked to the concurrent decrease in the intensity of the band associated with carbonate groups in the BG-700 sample, suggesting a substitution of CO_3_^2–^ groups by PO_4_^2–^ groups within the hydroxyapatite structure, which is characteristic of a B-type carbonated apatite.

With the increase in immersion time in SBF, the intensity of the band at 876 cm^–1^, associated with calcite, gradually decreases, indicating its progressive dissolution in the slightly basic SBF environment. This process releases calcium and carbonate ions into the solution, which subsequently participate in the nucleation and growth of a carbonated apatite layer onto the BG-700 surface. Concurrently, the emergence and progressive sharpening of the phosphate ν_4_(PO_4_^3–^) bands at 560 and 600 cm^–1^ confirm the progressive formation of the apatite phase. These observations are fully consistent with the XRD results, which reveal a clear structural evolution of the BG-700 sample: the initial calcite reflections decrease in intensity, while diffraction peaks characteristic of carbonated hydroxyapatite gradually emerge and become more defined. Together, the FTIR and XRD analyses provide complementary evidence that calcite acts as a transient, soluble phase, facilitating the rapid development of a biomimetic carbonated apatite layer, which is critical for the material’s bioactivity and potential biological performance.

SEM images of BG-RT and BG-700 samples before and after immersion in SBF for 24, 48, and 72 h days are shown in Figures 8 and 9, respectively. Prior to immersion, both samples exhibited irregular surface morphologies. After 24 h in SBF, the BG-RT sample demonstrated the formation of spherical structures of various sizes, while the BG-700 sample displayed numerous small, needle-like aggregates. An increase in the size of the core–shell particles was observed after 48 h, attributed to the deposition of a hydroxycarbonate apatite (HCA) layer. By 72 h of immersion, a dense apatite layer had formed on the surface of both samples, promoting the bonding of individual particles into spherical aggregates. This dense layer, clearly visible in the micrographs, can be associated with the formation of hydroxyapatite.^67,68^

**Figure 8:**
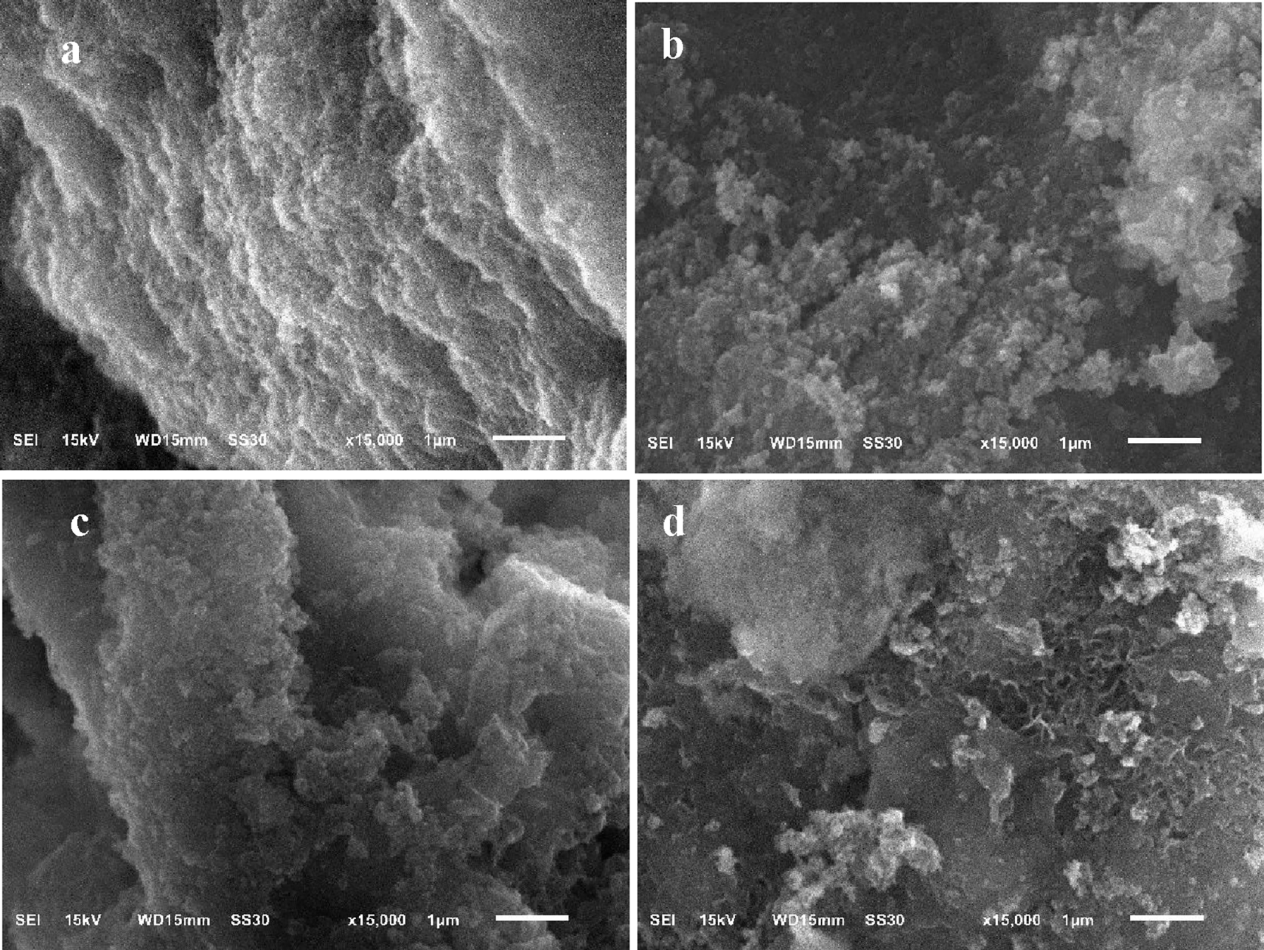
SEM micrographs of the BG-RT sample: (a) before (0 days) and after being soaked in SBF for (b) 24, (c) 48, and (d) 72 h.

**Figure 9:**
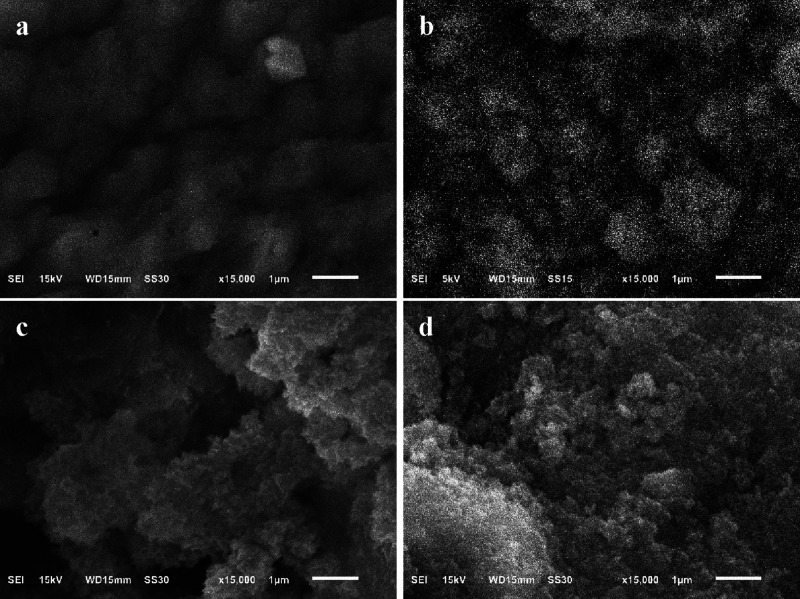
SEM micrographs of the BG-700 sample: (a) before (0 days) and after being soaked in SBF for (b) 24, (c) 48, and (d) 72 h.

### 3.3 Evaluation of Dental Treatments after Immersion in Artificial Saliva

Figure 10 presents SEM micrographs of dental samples subjected to treatment and artificial saliva immersion (Groups I, II, and III). The presence of well-defined dentinal tubules, with diameters typically on the order of 1–4 μm, is in agreement with the findings reported in the literature.^69^ In samples analyzed following a 2 day treatment period (see Figure 9a–c), the dentinal tubules were found to be fully patent.

**Figure 10:**
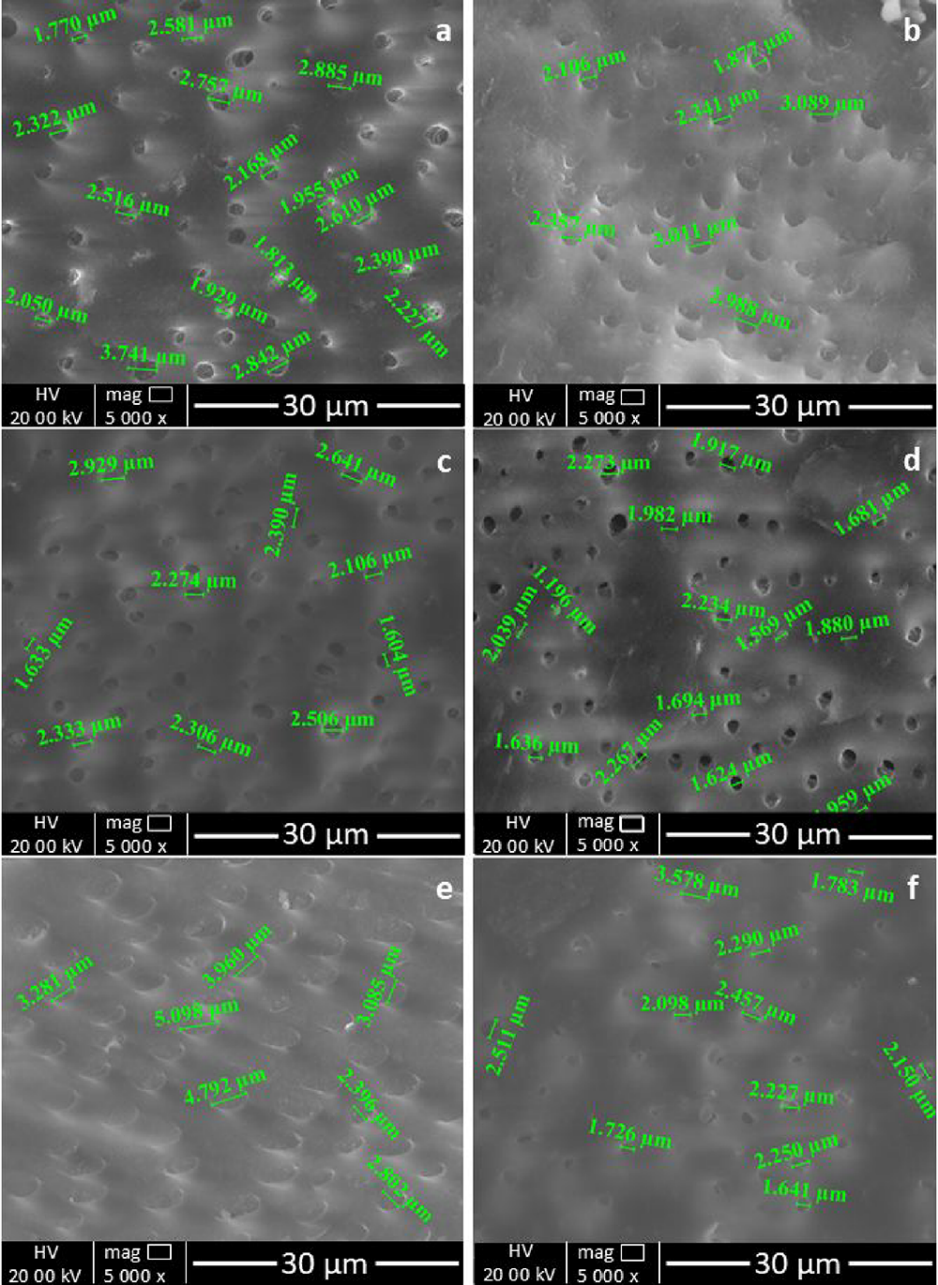
SEM micrographs of dental samples following treatments after immersion in artificial saliva: (a) Group I, 2 days; (b) Group II, 2 days; (c) Group III, 2 days; (d) Group I, 4 days; (e) Group II, 4 days; and (f) Group III, 4 days.

Upon examination of samples treated for 4 days, Figure 10d–f, significant morphological changes become apparent. Partial tubule occlusion has been observed in samples treated with based bioactive glass toothpaste (Group II) and commercial toothpaste (Group III), suggesting progressive remineralization with hydroxyapatite formation in the evaluated specimens.

Figure 11a displays the Raman spectra of samples from Groups I, II, and III, collected after 2 and 4 days of treatment with the experimental and commercial toothpastes (Groups II and III, respectively), as well as the control group (Group I). To enable direct comparison, the spectra were normalized to the 1448 cm^–1^ δ­(CH_2_) band, associated with collagen proteins in the dental structure.^70^

**Figure 11:**
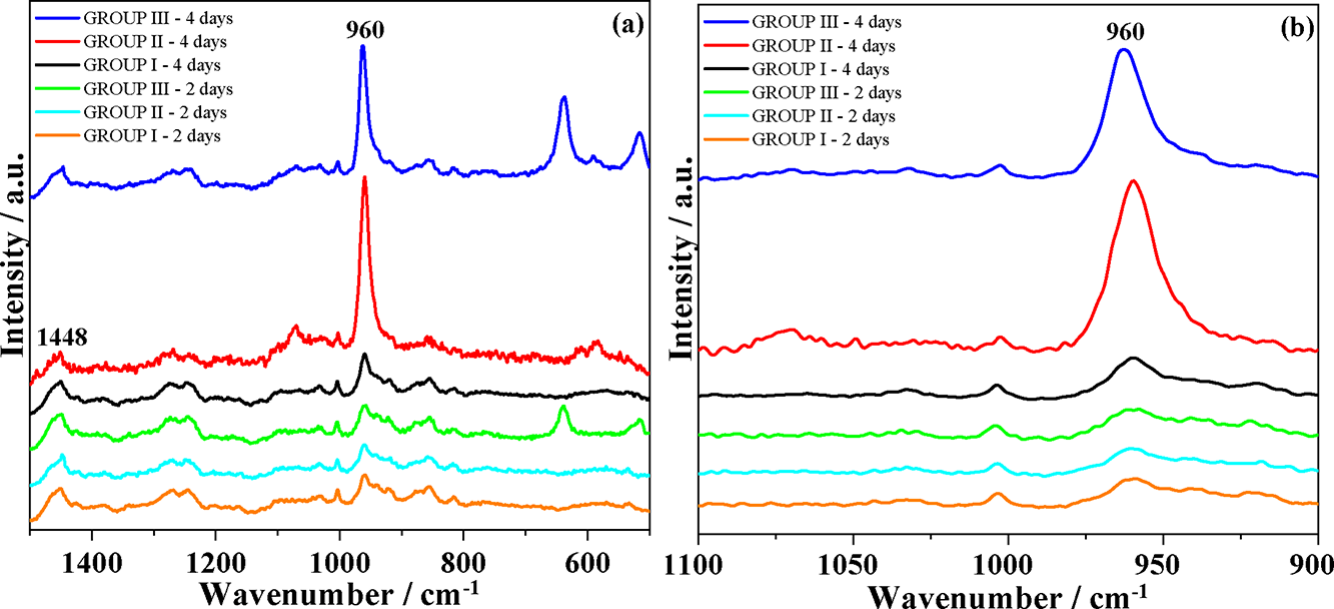
a) Normalized Raman spectra of dental samples from Groups I (control, without toothpaste application), II (treated with experimental toothpaste), and III (treated with commercial toothpaste) after 2 and 4 days of brushing treatment. (b) Magnification of the spectrum in the range of 900–1100 cm^–1^, highlighting the band at 960 cm^–1^.

No spectral changes were observed related to hydroxyapatite growth in samples subjected to only 2 days of treatment, represented by the 960 cm^–1^ ν_1_(PO_4_^3–^) band, as the Raman spectral profile remained similar to that of toothpaste-treated samples and the control, Figure 11b.^71,72^ However, after 4 days of treatment, significant spectral changes were detected, including an increase in the intensity of the ν_1_(PO_4_^3–^) band compared to the 2-day treated samples. Samples treated with toothpaste (Groups II and III) exhibited a more pronounced intensity enhancement, with the experimental toothpaste group showing the highest intensity increase. These results indicate that the BG toothpaste promoted more substantial hydroxyapatite growth on the tooth surface after 4 days compared to the commercial toothpaste.

### 3.4 Biological Evaluation

Data from the MTT assay showed that HaCat cells cultured in the BG-RT or BG-700 conditioned media presented high cell viability, i.e., similar to the control group grown in ideal conditions in all experimental times. Particularly at 7 days, the BG-RT group showed higher cell viability than the control group (*p* < 0.05) (Figure 12). These results align with previous reports showing that bioglasses are cytocompatible.^62,73^

**Figure 12:**
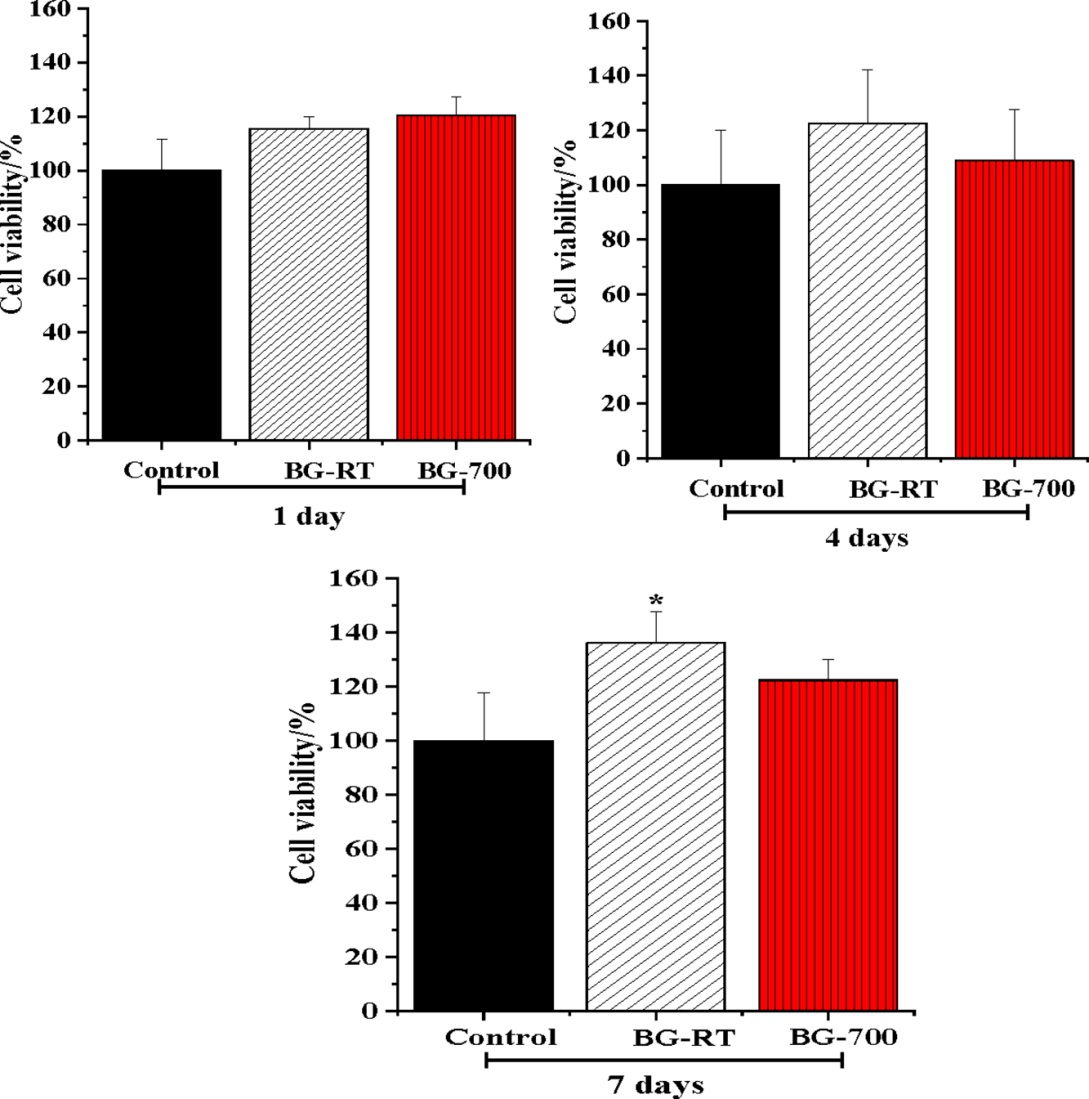
Cytocompatibility of BG-RT and BG-700 samples. Bar graph showing HaCat viability/proliferation upon contact with both samples conditioned medium for 1, 4, and 7 days. * means statistical difference between a group and the control (*p* < 0.05). Analysis of Variance (ANOVA) followed by Tukey post hoc.

## 4 Final Considerations

This study demonstrates that bioactive glasses synthesized via a modified sol–gel route using fumed silica can achieve excellent biological performance, even in the absence of high-temperature processing. The low-temperature material retained a fully amorphous structure and a remarkably high surface area, while calcination at 700 °C induced crystallization of calcite and reduced surface area, alongside a decrease in thermal stability. In spite of the structural and thermal discrepancies, both glasses demonstrated accelerated *in vitro* bioactivity, resulting in the formation of hydroxyapatite layers within 24 h in SBF. Furthermore, both glasses significantly enhanced HaCaT cell proliferation in comparison to the control. These findings highlight that the choice of thermal treatment offers a tunable pathway to control structure and properties without compromising biocompatibility, paving the way for more sustainable, energy-efficient approaches in the design of bioactive glasses for bone tissue regeneration.
